# Commissioning a clinical linac for preclinical research of heterogeneous intratumor irradiation with end‐to‐end tests

**DOI:** 10.1002/acm2.70402

**Published:** 2025-11-27

**Authors:** Shuting Wang, MengYao Li, Zhenkai Li, Xiaoying Fan, Yong Yin, Tianyuan Dai

**Affiliations:** ^1^ Department of Graduate Shandong First Medical University Shandong Academy of Medical Sciences Jinan China; ^2^ Department of Radiation Oncology Physics and Technology Shandong Cancer Hospital and Institute Shandong First Medical University and Shandong Academy of Medical Sciences Jinan China; ^3^ Department of Oncology Afliated Hospital of Southwest Medical University Luzhou Sichuan China

**Keywords:** clinical linac, conical collimators, end‐to‐end test, Heterogeneous intratumor irradiation (HII), small animal irradiation

## Abstract

**Background:**

Although it is not clear how the heterogeneous intratumor irradiation pattern affects the biological effect, investigations of the biological effect dependence on the dose and size of the peak region are essential in the current preclinical research.

**Purpose:**

This work develops and validates a cone‐equipped clinical linac platform for heterogeneous intratumor irradiation (HII) of small animals, making translationally relevant preclinical radiobiology studies accessible to more clinical centers.

**Methods:**

Output ratios (ORs) for cones (from 4 mm to 17.5 mm diameters) were measured using Gafchromic EBT3 films on a Varian TrueBeam linac (6 MV FFF beam). Tissue maximum ratios (TMR) and off‐axis ratios (OAR) were incorporated into the Eclipse treatment planning system (TPS) to configure the beam model. End‐to‐end test was performed using both a mouse phantom and tumor‐bearing mice, with dose distributions verified via film dosimetry and *γ* analysis (3%/3 mm and 3%/2 mm criteria).

**Results:**

The system achieved millimeter‐scale precision, with *γ* pass rates ≥ 99% (3%/2 mm). Dose distributions confirmed the feasibility of delivering heterogeneous doses (e.g., 14 Gy via 4 mm cone + 2 Gy via 15 mm cone) while sparing normal tissues.

**Conclusion:**

This study established a robust preclinical HII platform using clinical linac infrastructure, enabling precise dose delivery for investigating dose‐response relationships and supporting future translational research.

## 1 INTRODUCTION

Heterogeneous intratumor irradiation (HII) delivers heterogeneous dose distributions to tumor volumes, selectively targeting subregions with high doses while sparing adjacent normal tissues.^1^, ^2^, ^3^, ^4^, ^5^ Clinically, HII has been implemented using physical GRID blocks mounted on the linac or lattice therapy plan modulated through multi‐leaf collimators (MLCs).^6^, ^7^, ^8^, ^9^, ^10^ Although not yet a mainstream treatment modality, these kinds of radiation therapy, called spatially fractionated radiation therapy (SFRT), have demonstrated promising dosimetric and clinical outcomes in radioresistant, deep‐seated, and bulky tumors.^11^, ^12^, ^13^, ^14^, ^15^, ^16^, ^17^, ^18^, ^19^

Conical collimators (cones), commonly used in stereotactic radiosurgery (SRS), provide an alternative approach for HII delivery. These accessories attach to the linac head with dedicated mounting systems, ensuring precise and reproducible central axis alignment while defining small, circular radiation fields.^20^ Cones offer several advantages, including high‐dose delivery, customizable field sizes, and superior beam‐shaping capabilities. Leveraging these dosimetric and physical properties, we hypothesized that cones could be adapted to design spatially fractionated treatment plans suitable for preclinical murine models.

In this study, the HII preclinical research plan was delivered using commercial cones mounted on a Varian TrueBeam linac. Due to the challenges of small‐field dosimetry, such as variations in stopping‐power ratios and perturbation factors, that is field‐ and detector‐specific output correction factors (e.g., IAEA TRS‐483 recommendations) must be applied to ensure accurate dose measurements.^21^ Several methodologies for determining output ratios (ORs) for small fields (e.g., 5 mm cones) have been reported, including those by Cheng et al., Smith et al., and Oliver et al.^22^, ^23^, ^24^ However, discrepancies may arise due to differences in detector selection, cone/linac models, treatment planning system (TPS) algorithms, and correction factor application. The primary objective of this work was to evaluate the dosimetric accuracy and preclinical reliability of Cone‐based HII preclinical research for tumor irradiation of small animals.

## 2 METHODS

### 2.1 Beam data measurements and beam model commissioning

The Varian TrueBeam linear accelerator (linac) was calibrated to deliver 1 cGy per monitor unit (MU) for a 10 × 10 cm^2^ reference field at 100 cm source‐to‐surface distance (SSD) and depth of dose maximum (d_max_). For the film calibration process, Gafchromic EBT3 films (Ashland Advanced Materials, Bridgewater, New Jersey) were cut into rectangular pieces of 3 × 20 cm^2^. The films were positioned 5 cm underwater with an SSD of 100 cm and a field size of 10 × 10 cm^2^. Seven dose values (0.5, 1, 2, 4, 8, 16, and 20 Gy) were delivered to generate a calibration curve. Unirradiated films were retained to validate the calibration. After a 24‐hour waiting period to ensure color stability, the films were scanned using an Epson Expression 13000XL flatbed scanner. The measurements were analyzed with the RIT 113 software program.

**FIGURE 1:**
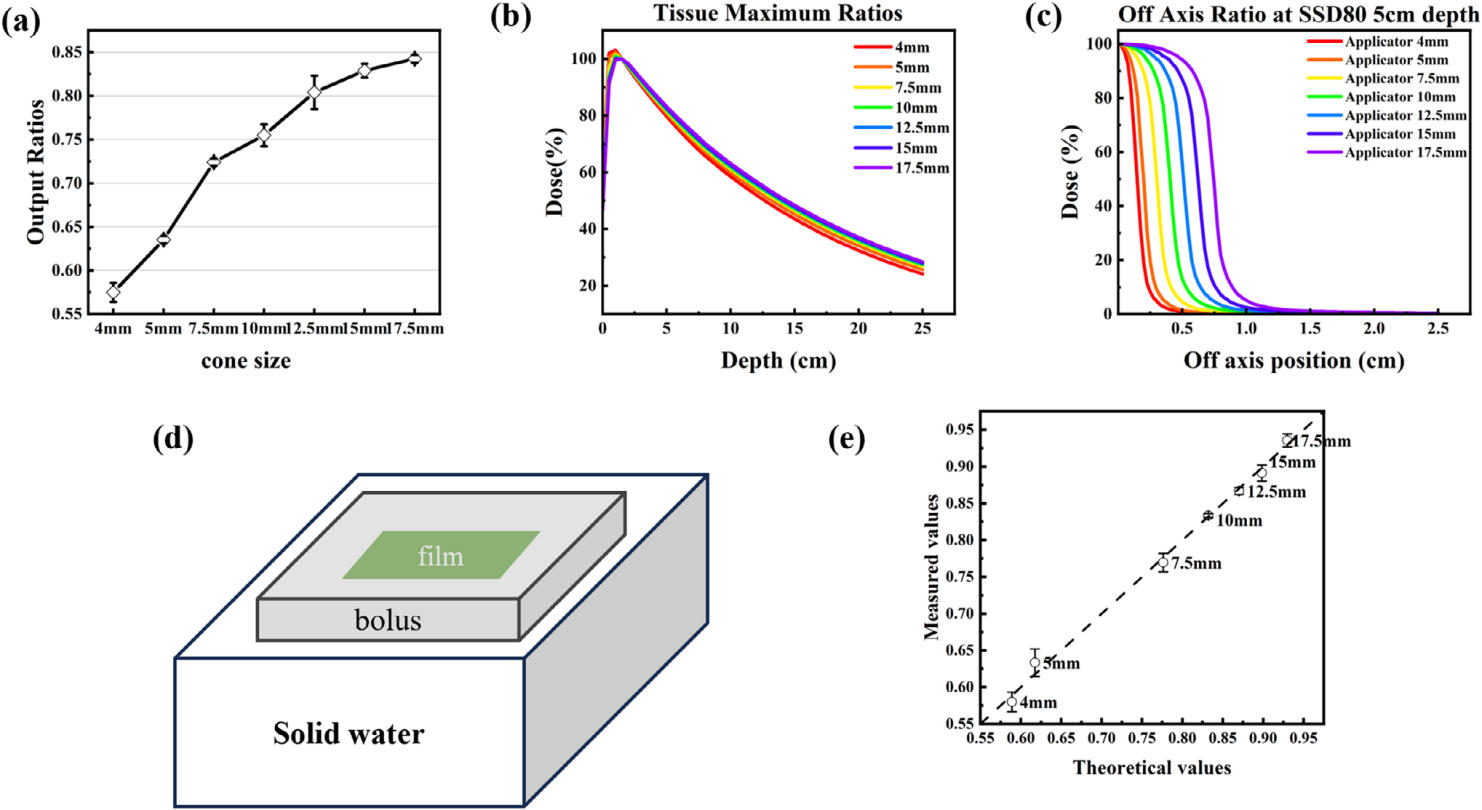
(a) EBT3 film‐measured ORs for cones in different diameters. (b) TMR data with depth in mm on the *x*‐axis for different cones. (c) OAR data with off‐axis position in mm on the *x*‐axis for various cones. (d) Schematic diagram of beam model validation with a homogeneous phantom with different cones, in which the film under the bolus is displayed. (e) Results of beam model validation with a homogeneous phantom for various cones.

Commissioning data were acquired using Gafchromic EBT3 film scanned with an Epson Expression 13000XL flatbed scanner for a 6 MV flattening filter‐free (6FFF) and a dose rate of 1200 MU/min. For cone measurements, the jaw size was set to 5 × 5 cm^2^ in accordance with Varian's Golden Beam Data (GBD) recommendations. The ORs were measured at SSD of 95 cm and a depth of 5 cm, with normalization performed relative to the 10 × 10 cm^2^ reference field under identical measurement conditions. All measurements were repeated three times. The ORs are presented as mean values with error bars representing the standard deviation. Film digitization was conducted using the Epson SCAN 2 software in 48‐bit red, green, and blue (RGB) uncompressed transmission mode at a scanning resolution of 300 dots per inch (dpi), with all post‐processing image corrections disabled. Dose extraction from the scanned films was performed using RIT 113 software (Radiological Imaging Technology), with the central dose within the target region recorded for analysis. To minimize measurement uncertainties associated with film positioning, all films were consistently placed at a fixed location on the scanner bed and oriented longitudinally during scanning.^25^ Tissue maximum ratios (TMR) and off‐axis ratios (OAR) (Figure 1c, d) were cited for small fields21. The Eclipse collapsed cone convolution (CDC) algorithm was configured using measured ORs, TMR, and OARs data.

### 2.2 Beam model validation with homogeneous phantom

The homogeneous phantom validation was performed to simulate the actual irradiation conditions of mice. Films were placed on the surface of solid water, with a 1 cm thick bolus covering the films (Figure 1d), and irradiated with 600 MU of x‐rays using cones of different sizes. This procedure was repeated three times. The cone collimator factor is defined as the ratio of the peak dose when using a cone collimator at a specific field size to the dose under the accelerator output calibration condition (where 1 MU delivers 1.0 cGy).

### 2.3 End‐to‐end test for preclinical test

As a validation of the application of clinical linac on the small animal irradiation for preclinical HII research, a 3D‐printed mouse phantom was developed, and end‐to‐end tests were conducted based on the CT simulation of the mouse phantom with a single‐layer EBT3 film insert. In the Eclipse system, a representative treatment plan was developed using 4 and 15 mm cones. The 4 mm cone was used to deliver a dose of 14 Gy to the peak region. The 15 mm cone was used to irradiate the whole tumor with 2 Gy. As a result, this representative plan achieved a 16 Gy dose to the peak region and a 2 Gy dose to other parts of the tumor. This simulated the scenario of HII preclinical research to investigate the biological effect dependence on the dose and size of the peak region, where the 16 Gy high‐dose region is to induce tumor‐specific immunogenic response and the 2 Gy low‐dose region is to reprogram the immunosuppressive tumor microenvironment. These plans irradiated the aforementioned mouse phantom with SSD of 99 cm, and the distance from the skin on the central axis of the field to the film placement position was 1 cm. Subsequently, three cross‐sectional plane dose distributions at a depth of 1 cm were generated. The RIT 113 software was used to compare these dose planes with the 2D dose distributions obtained from film dosimetry under the same conditions. The criteria applied for γ analysis included 3%/3 and 3%/2 mm.

### 2.4 In‐vivo dose verification

**FIGURE 2:**
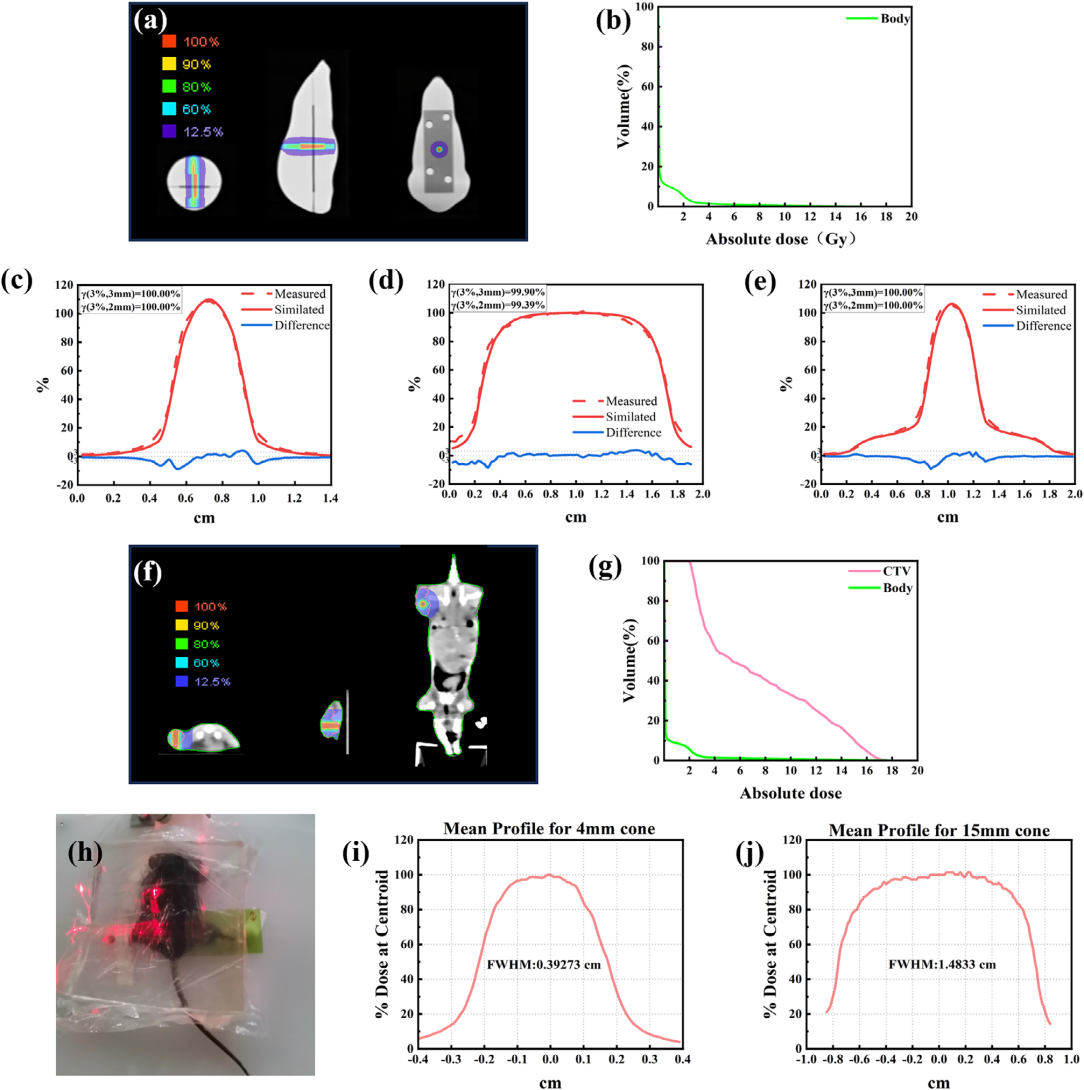
(a) Dose distributions in orthogonal views and (b) dose volume histogram (DVH) of a mouse phantom irradiated with two cones (4 mm, 14 Gy and 15 mm, 2 Gy). Comparisons between film‐measured and simulated dose profiles within the mouse phantom for the 4 mm cone (c), 15 mm cone (d), and sequential irradiation with the 4 + 15 mm cones (e). Blue lines represent the percentage residuals between the measured and simulated doses. (f) Dose distributions in orthogonal views and (g) dose volume histograms of a mouse irradiated with two cones (4 and 15 mm). (h) Mouse positioning during actual irradiation, along with the corresponding dose distributions (i and j).

To validate the preclinical application of Cone‐HII, dose verification under real irradiation conditions was performed using tumor‐bearing mice based on CT simulation and a validated beam model. C57BL/6J mice with subcutaneous MC38 tumors were used for this study. The mice were scanned using a Siemens CT scanner. Contours, including the clinical target volume (CTV), were delineated on the CT image sets. The image datasets were then imported into the Eclipse TPS for plan creation. Further validation irradiation experiments were conducted in this real tumor‐bearing mouse model to evaluate the dose distribution characteristics of the 4 and 15 mm collimators under in vivo conditions. Two treatment plans were developed to simulate the actual treatment process in mice: one plan delivered a 14 Gy dose using a 4 mm collimator, and the other delivered a 2 Gy dose using a 15 mm collimator. Mouse positioning followed the same protocol as in the phantom validation, as illustrated in Figure 2h. The mean dose distribution profiles of the films were analyzed to complete the validation.

## 3 RESULTS

### 3.1 Conal dosimetry

The ORs for conical collimators of varying diameters (ranging from 4 to 17.5 mm) were measured using Gafchromic EBT3 films under a 6 MV FFF beam, as illustrated in Figure 1a. The results demonstrate a clear trend of decreasing ORs with decreasing cone diameter. Figure 1b presents the tissue maximum ratios (TMR) for different cones as a function of depth. The TMR curves show a rapid dose falloff beyond the depth of maximum dose (d_max_), particularly for smaller cones, due to the increased scatter loss and reduced photon fluence in narrow beams. The larger cones (e.g., 17.5 mm) exhibit more gradual attenuation, resembling conventional field characteristics. OAR depicted in Figure 1c illustrates the beam profiles for various cones. The profiles exhibit sharp penumbra and high dose gradients, especially for smaller cones, which is critical for achieving precise spatial fractionation. The 4 mm cone shows the narrowest beam width and most rapid dose falloff at the edges, confirming its suitability for creating high‐dose peak regions within heterogeneous irradiation patterns. Figure 1e summarizes the results of the beam model validation using a homogeneous solid water phantom. The measured cone output factors agree closely with the values configured in the Eclipse TPS. The consistency across repeated measurements also underscores the reproducibility of the cone‐based delivery system.

### 3.2 Commissioning beam data and end‐to‐end test

Figure 2a shows the dose map of the dose distribution of cone‐based HII in the mouse phantom. This dose map demonstrates how cones of different sizes achieve low‐dose and high‐dose regions. Figure 2b displays the DVH curve of the mouse phantom's body. Figures 2c–e show the *γ* analysis pass rates based on the criteria of 3%/3 and 3%/2 mm in the RIT 113 software for the dose planes measured with EBT3 films and the dose planes of each end‐to‐end plan. End‐to‐end measurements in the mouse phantom revealed no significant issues in the clinical workflow or dose calculation for the 4 and 15 mm collimators. All plans achieved a 99% pass rate under the strictest analysis criteria (Figures 2c–e).

Figure 2f presents the dose map of the dose distribution of cone‐based HII in the mouse. Figure 2g is the DVH curve of the mouse. The results show that 99.57% of the volume received a dose of 2 Gy, and 4.79% of the volume received a dose of 16 Gy.

In the validation irradiation experiments using real tumor‐bearing mouse models, the radiation beam profiles of both collimator sizes exhibited stable dose distribution characteristics (as shown in Figures 2i,j). The results were consistent with the dose distribution trends obtained in phantom validation, which further verified the accuracy and reliability of dose delivery by the two collimators in in vivo tumor irradiation. This provides an experimental basis for the design of precise radiotherapy schemes for tumor‐bearing mice in subsequent preclinical studies.

## 4 DISCUSSION

We developed an MV‐level HII platform for preclinical research using a clinical TrueBeam linac and cone collimators. The system achieved millimeter‐scale precision, with *γ* pass rates ≥99% (3%/2 mm criteria). The system was rigorously validated for dosimetric accuracy, plan delivery reliability, and in vivo irradiation, overcoming the limitations of conventional small‐animal irradiators and GRID blocks.

Under the γ criterion of 3%/2 mm in RIT113 film analysis, all treatment plans achieved a passing rate ≥99%, confirming the integrity of the data chain between the Eclipse planning system and TrueBeam, as well as the millimeter‐scale mechanical precision required for spatially fractionated stereotactic irradiation. By flexibly combining cone collimators of different apertures, steep dose gradients were generated in mice, enabling a clinically relevant HII pattern of “high‐dose target coverage with minimal peripheral dose.” This provides a controlled and reproducible experimental setup for dose‐response investigations.

Unlike conventional small‐animal irradiators, this platform utilizes clinical MV beams, avoiding the rapid depth‐dose falloff and large penumbra associated with low‐energy x‐rays. Compared to fixed‐aperture GRID blocks, the cone collimators allow rapid and precise mounting on the gantry while enabling adjustable peak‐to‐valley dose ratio (PVDR) through customizable aperture sizes, spacing, and MU modulation. This flexibility facilitates comprehensive investigations into how HII parameters influence the dose‐immunity‐efficacy relationship, thereby generating valuable evidence for future clinical translation.

## 5 CONCLUSION

Phantom and in vivo validations confirmed the accuracy of cone‐based HII for preclinical applications, providing a foundation for dose‐response studies and future clinical translation. These results provide a robust dosimetric foundation for subsequent preclinical investigations, including dose‐response relationship studies, radiosensitizer evaluation, and combination immunotherapy research. Furthermore, this work establishes essential technical and data‐driven groundwork for future advancements toward cutting‐edge directions such as smaller radiation fields, ultra‐high dose rates, and FLASH radiotherapy.

## AUTHOR CONTRIBUTIONS

*Conceived the study, participated in its design, carried out data acquisition and analysis, and drafted the manuscript*: Shuting Wang. *Contributed to data collection and experimental execution*: Zhenkai Li and MengYao Li. *Assisted with data interpretation and manuscript revision*: Xiaoying Fan. *Supervised the project, provided critical intellectual input, and revised the manuscript extensively*: Yong Yin and Tianyuan Dai. All authors read and approved the final version of the manuscript.

## CONFLICT OF INTEREST STATEMENT

The authors declare that they have no competing interests.

## ETHICS STATEMENT

This study was approved by the Research Ethics Board of the Shandong Cancer Hospital (approval no: 202509105). Written informed consent was waived by the Institutional Review Board.

## Data Availability

All data included in this study are available upon request by contacting the corresponding author.

## References

[1] Mohiuddin M , Curtis DL , Grizos WT , Komarnicky L . Palliative treatment of advanced cancer using multiple nonconfluent pencil beam radiation. A pilot study. Cancer. 1990;66(1):114‐118. doi:10.1002/1097-0142(19900701)66:1<114::AID-CNCR2820660121>3.0.CO;2-L 1693874

[2] Reiff JE , Huq MS , Mohiuddin M , Suntharalingam N . Dosimetric properties of megavoltage grid therapy. Int J Radiat Oncol Biol Phys. 1995;33(4):937‐942. doi:10.1016/0360-3016(95)00114-3 7591906

[3] Mohiuddin M , Fujita M , Regine WF , Megooni AS , Ibbott GS , Ahmed MM . High‐dose spatially‐fractionated radiation (GRID): a new paradigm in the management of advanced cancers. Int J Radiat Oncol Biol Phys. 1999;45(3):721‐727. doi:10.1016/S0360-3016(99)00170-4 10524428

[4] Bergeron P , Milliat F , Deutsch E , Mondini M . Heterogeneous intratumor irradiation: a new partner for immunotherapy. Oncoimmunology. 2024;13(1):2434280. doi:10.1080/2162402X.2024.2434280 39589158 PMC11601051

[5] Jagodinsky JC , Vera JM , Jin WJ , et al. Intratumoral radiation dose heterogeneity augments antitumor immunity in mice and primes responses to checkpoint blockade. Sci Transl Med. 2024;16(765):eadk0642. doi:10.1126/scitranslmed.adk0642 39292804 PMC11522033

[6] Buckey C , Stathakis S , Cashon K , et al. Evaluation of a commercially‐available block for spatially fractionated radiation therapy. J Appl Clin Med Phys. 2010;11(3):3163. doi:10.1120/jacmp.v11i3.3163 20717082 PMC5720442

[7] Ha JK , Zhang G , Naqvi SA , Regine WF , Yu CX . Feasibility of delivering grid therapy using a multileaf collimator. Med Phys. 2006;33(1):76‐82. doi:10.1118/1.2140116 16485412

[8] Neuner G , Mohiuddin MM , Vander Walde N , et al. High‐dose spatially fractionated GRID radiation therapy (SFGRT): a comparison of treatment outcomes with Cerrobend vs. MLC SFGRT. Int J Radiat Oncol Biol Phys. 2012;82(5):1642‐1649. doi:10.1016/j.ijrobp.2011.01.065 21531514

[9] Meigooni AS , Dou K , Meigooni NJ , et al. Dosimetric characteristics of a newly designed grid block for megavoltage photon radiation and its therapeutic advantage using a linear quadratic model. Med Phys. 2006;33(9):3165‐3173. doi:10.1118/1.2241998 17022209

[10] Stathakis S , Esquivel C , Gutiérrez AN , Shi C , Papanikolaou N . Dosimetric evaluation of multi‐pattern spatially fractionated radiation therapy using a multi‐leaf collimator and collapsed cone convolution superposition dose calculation algorithm. Appl Radiat Isot. 2009;67(10):1939‐1944. doi:10.1016/j.apradiso.2009.06.012 19632125

[11] Huhn JL , Regine WF , Valentino JP , Meigooni AS , Kudrimoti M , Mohiuddin M . Spatially fractionated GRID radiation treatment of advanced neck disease associated with head and neck cancer. Technol Cancer Res Treat. 2006;5(6):607‐612. doi:10.1177/153303460600500608 17121437

[12] Peñagarícano JA , Moros EG , Ratanatharathorn V , Yan Y , Corry P . Evaluation of spatially fractionated radiotherapy (GRID) and definitive chemoradiotherapy with curative intent for locally advanced squamous cell carcinoma of the head and neck: initial response rates and toxicity. Int J Radiat Oncol Biol Phys. 2010;76(5):1369‐1375. 19625138 10.1016/j.ijrobp.2009.03.030

[13] Zhang H , Wang JZ , Mayr N , et al. Fractionated grid therapy in treating cervical cancers: conventional fractionation or hypofractionation?. Int J Radiat Oncol Biol Phys. 2008;70(1):280‐288. doi:10.1016/j.ijrobp.2007.08.024 17967516

[14] Meigooni AS , Parker SA , Zheng J , Kalbaugh KJ , Regine WF , Mohiuddin M . Dosimetric characteristics with spatial fractionation using electron grid therapy. Med Dosim. 2002;27(1):37‐42. doi:10.1016/S0958-3947(02)00086-9 12019964

[15] Zwicker RD , Meigooni A , Mohiuddin M . Therapeutic advantage of grid irradiation for large single fractions. Int J Radiat Oncol Biol Phys. 2004;58(4):1309‐1315. doi:10.1016/j.ijrobp.2003.07.003 15001276

[16] Peng V , Suchowerska N , Rogers L , Claridge Mackonis E , Oakes S , McKenzie DR . Grid therapy using high definition multileaf collimators: realizing benefits of the bystander effect. Acta Oncol. 2017;56(8):1048‐1059. doi:10.1080/0284186X.2017.1299939 28303745

[17] Griffin RJ , Ahmed MM , Amendola B , et al. Understanding high‐dose, ultra‐high dose rate, and spatially fractionated radiation therapy. Int J Radiat Oncol Biol Phys. 2020;107(4):766‐778. doi:10.1016/j.ijrobp.2020.03.028 32298811

[18] Peñagarícano JA , Griffin R , Corry P , Moros E , Yan Y , Ratanatharathorn V . Spatially fractionated (GRID) therapy for large and bulky tumors. J Ark Med Soc. 2009;105(11):263‐265. 19475814

[19] Mohiuddin M , Park H , Hallmeyer S , Richards J . High‐Dose radiation as a dramatic, immunological primer in locally advanced melanoma. Cureus. 2015;7(12):e417. 26848410 10.7759/cureus.417 PMC4725734

[20] International commission on radiation units and measurements. J icru. 2014;14(1):Np. 10.1093/jicru/ndw040 27789602

[21] Palmans H , Andreo P , Huq MS , Seuntjens J , Christaki KE , Meghzifene A . Dosimetry of small static fields used in external photon beam radiotherapy: summary of TRS‐483, the IAEA‐AAPM international code of practice for reference and relative dose determination. Med Phys. 2018;45(11):e1123‐e1145. doi:10.1002/mp.13208 30247757

[22] Cheng JY , Ning H , Arora BC , Zhuge Y , Miller RW . Output factor comparison of Monte Carlo and measurement for Varian TrueBeam 6 MV and 10 MV flattening filter‐free stereotactic radiosurgery system. J Appl Clin Med Phys. 2016;17(3):100‐110. doi:10.1120/jacmp.v17i3.5956 27167266 PMC5690931

[23] Smith CL , Montesari A , Oliver CP , Butler DJ . Evaluation of the IAEA‐TRS 483 protocol for the dosimetry of small fields (square and stereotactic cones) using multiple detectors. J Appl Clin Med Phys. 2020;21(2):98‐110. doi:10.1002/acm2.12792 PMC7021012 31886615

[24] Oliver CP , Butler DJ , Takau V , Williams I . Survey of 5 mm small‐field output factor measurements in Australia. J Appl Clin Med Phys. 2018;19(2):329‐337. doi:10.1002/acm2.12259 29368796 PMC5849830

[25] Ruiz‐Morales C , Antonio Vera‐Sánchez J , González‐López A . Optimizing the recalibration process in radiochromic film dosimetry. Phys Med Biol. 2020;65(1):015016. doi:10.1088/1361-6560/ab58dd 31746787

